# The Royal Hospital for Incurables

**Published:** 1915-05-15

**Authors:** 

**Affiliations:** President, Ladies' Association. Margaret Loch, Hon. Treasurer.


### THE EDITOR'S LETTER-BOX.

### The Royal Hospital for Incurables.

*To the Editor of* The Hospital.

Sir,—At this moment, when the war is making such large demands on the generosity of the public, many of the older charities are feeling acutely the need of help. Of these, none makes a more urgent appeal to our sympathies than those which deal with incurable illness. War or no war, these most pathetic cases continue to exist. The Ladies' Association of the Royal Hospital for Incurables, Putney Heath, helps entirely unbefriended and therefore specially pitiable cases. The sum of £250 is at present urgently needed to secure the election, on May 28, of several totally unbefriended cases. This year it would be useless to attempt raising it by means of an entertainment of any kind. Will the public help us?

All contributions to be sent to the Hon. Secretary, 93 Elizabeth Street, Eaton Square, S.W.

Helena, President, Ladies' Association.
Margaret Loch, Hon. Treasurer.

